# Cross-species validation of human specific STR system, *SureID*^*®*^*21G* and *SureID*^*®*^*23comp* (Health Gene Technologies) in Chimpanzee (*Pan Troglodytes*)

**DOI:** 10.1186/s13104-019-4801-3

**Published:** 2019-11-19

**Authors:** Abhishek Singh, Mukesh Thakur, Vivek Sahajpal, Sujeet K. Singh, Kailash Chandra, Arun Sharma, Nisha Devi, Ausma Bernot

**Affiliations:** 1 Zoological Survey of India, New Alipore, Kolkata, West Bengal 700 053 India; 2 State Forensic Science Laboratory, Directorate of Forensics Services, Junga, Shimla, Himachal Pradesh 171218 India; 3 Ningbo Health Gene Technologies Co. Ltd., Ningbo, 315040 People’s Republic of China

**Keywords:** SureID^®^ 21G, SureID^®^ 23comp, Cross reactivity, Chimpanzees, Human identification

## Abstract

**Objectives:**

The human specific commercially available STRs system are often not tested in non human primates for their cross applicability. The aim of this study is to test Cross-species validation of two commercially available human specific STR kits i.e. SureID^®^ 21G and SureID^®^ 23comp (Health Gene Technologies) for their positive application in Chimpanzee (*Pan troglodytes*).

**Result:**

In SureID^®^ 21G, 19 loci amplified and while 20 loci amplified in SureID^®^ 23comp. All the amplified loci in both STR kits were found polymorphic and the locus Amelogenin showed differential banding patterns between male and female revealing their known gender. The present study validates the applicability of these human specific STR kits in Chimpanzee that can be used in forensics analysis, paternity testing and population genetic studies.

## Introduction

Human identification kits based on Short Tandem Repeats (STRs) markers are widely accepted and utilized in several fields of population genetics and forensic case works. Human identification prior to the development of genetic methods was preferably attempted by the serological techniques as they have proven in successfully determining the species of origin through a presumptive test in forensic cases [1]. However, there have been several instances where human antiserum showed cross-reactivity with non-human blood. For example, Landsteiner and Miller [2] documented that the anthropoid blood of Gorilla, Orangutan and Chimpanzee showed precipitation with human antiserum. This indicated that serological tests showed less specificity and sensitivity with the phylogenetically close relatives of human. Numerous STR panels are used for human identification in forensics analysis and population genetic studies [3–5] but none of the marker systems has been validated for its cross applicability in non-human primate except PowerPlex^®^ 21 human identification System (Promega, USA) [6]. However, it is important to explore the possibility of cross-species amplification of these marker systems with non-human primates, so as to understand its applicability and link the specific use (possibility of exclusion in cases where samples of human and non-human primates are mixed).

We attempted to check the cross-reactivity of two human-specific STR marker system SureID^®^ 21G and SureID^®^ 23comp (Health Gene Technologies) in Chimpanzee following the Thakur et al. [6] which has established applicability of PowerPlex^®^ 21 human identification System (Promega, USA) in Chimpanzee.

## Main text

### Methods

#### Sample collection, DNA extraction and genotyping

Samples from three chimpanzees (two males and one female) were received from the Alipore Zoological Garden, Kolkata. These individuals were young, seems to be about two to three old in age and were confiscated by the Officials of Wildlife Crime Control Bureau, Eastern Unit. The Alipore Zoological Garden, Kolkata did not have any other supplementary information to declare about these confiscated animals. Genotyping of chimpanzees DNAs was carried out using two newly available human STR identification kit i.e. SureID^®^ 21G and SureID^®^ 23comp (Health Gene Technologies). The SureID^®^ 21G includes 20 autosomal STR markers (CSF1PO, D13S317, D16S539, D18S51, D19S433, D5S818, D12S391, D1S1656, D21S11, D2S1338, D3S1358, D6S1043, D7S820, D8S1179, FGA, Penta D, Penta E, TH01, TPOX, vWA) and with one gender determination marker Amelogenin (AMEL) whereas the SureID^®^ 23comp comprises of 22 STR loci (D18S1364, D1S1656, D13S325, D5S2800, D9S1122, D4S2366, D3S1744, D12S391, D11S2368, D21S2055, D20S480, D8S1132, D7S3048, D2S441, D19S253, D10S1248, D17S1301, D22-GATA198B05, D16S539, D6S474, D14S1434, D15S659) and one gender determination locus amelogenin (AMEL). The separate PCR reactions were carried out on GeneAmp PCR system 9700 thermocycler (Applied Biosystems, USA) in a total volume of 25 µl separately with SureID^®^ 21G PCR reagents (PCR Master mix, SureID^®^ 21G primer mix, DNase/RNase-Free water) and with SureID^®^ 23comp PCR reagents (PCR Master mix, SureID^®^ 23comp, DNase/RNase-Free water) following manufacturers protocols. Positive and negative controls were used throughout the reaction as per the kit guidelines. The amplification products were run on the ABI 3130 Genetic analyzer (Applied Biosystems, USA) for fragment analysis.

#### Statistical analysis

Allele calling was manually done using GeneMapper ID version 3.2 (Applied Biosystems, USA). The allelic data was exported into the Microsoft Excel and scoring and re-arrangement of the sizes of alleles were performed for the assessment of genetic diversity indices. Software, GENEALEX version 6.5 [7] was used to estimate the genetic diversity indices such as the observed number of alleles per locus (Na), an effective number of alleles (Ne), Observed heterozygosity (Ho), Expected heterozygosity (He) and Inbreeding Coefficient (F). Allelic data was used in estimating the genealogical relationship or relatedness among the three chimpanzees of unknown ancestry. The computer program, ML-Relate that calculates maximum likelihood estimates of relatedness and relationship was used [8] which does accommodate null alleles which may arise when using heterologous markers.

### Results

#### Applicability of SureID^®^ 21G Human STR Identification kit

Of the 21 loci in SureID^®^ 21G Human STR Identification kit, two loci i.e. D12S391 & D21S11 did not amplify in all three chimpanzee samples and other 19 loci were polymorphic. The detail allelic data can be obtained upon request. The locus AMEL correctly assign the sexes of known chimpanzees providing differential banding patterns in males and females (single allele of 112 bp in female and 112, 117 bp alleles in males). This exhibited discrimination power of SureID^®^ 21G Human STR Identification systems in determining genders of Chimpanzee. In total, 62 alleles were found associated with 19 loci. The number of observed alleles ranged from 6 (D1S1656 & D2S1338) to 1 (D5S818) with 3.26 ± 0.30 mean number of alleles per locus. The effective number of alleles with mean 2.84 ± 0.31 did not exceed the observed number of alleles for all loci. The mean Ho and He were 0.63 ± 0.07 and 0.57 ± 0.05 with mean UHe of 0.68 ± 0.06. A negative mean F value of − 0.13 ± 0.09 were observed representing a high heterozygosity than expected (Table 1). Out of 19 loci, 8 loci were observed relatively more heterozygous with ≥ 4 alleles/locus indicating that the analyzed chimpanzees plausibly belong to unrelated genetic background. The genealogical relationship between the individuals based on four possible categories i.e. unrelated (U), half-siblings (HS), full-siblings (FS) and parent-offspring (PO) for 18 loci i.e. AMEL, CSF1PO, D13S317, D16S539, D18S51, D19S433, D1S1656, D2S1338, D3S1358, D6S1043, D7S820, D8S1179, FGA, Penta D, Penta E, TH01, TPOX and vWA (which amplified in all three chimpanzees) suggested that analyzed individuals were genetically unrelated (LnL(R) − 62.26 to − 58.17; Table 2).

**Table 1:** Genetic diversity estimates of Chimpanzees with SureID^®^ 21G (Health Gene Technologies) human STR Identification kit

Locus	Na	Ne	Ho	He	uHe	F
AMEL	2.000	1.800	0.667	0.444	0.533	− 0.500
CSF1PO	2.000	1.800	0.667	0.444	0.533	− 0.500
D13S317	3.000	2.571	1.000	0.611	0.733	− 0.636
D16S539^a^	4.000	3.600	0.667	0.722	0.867	0.077
D18S51	2.000	2.000	0.333	0.500	0.600	0.333
D19S433^a^	4.000	3.600	0.333	0.722	0.867	0.538
D1S1656^a^	6.000	6.000	1.000	0.833	1.000	− 0.200
D2S1338^a^	6.000	6.000	1.000	0.833	1.000	− 0.200
D3S1358	3.000	2.571	1.000	0.611	0.733	− 0.636
D5S818	1.000	1.000	0.000	0.000	0.000	#N/A
D6S1043^a^	4.000	3.000	0.667	0.667	0.800	0.000
D7S820	3.000	2.571	0.667	0.611	0.733	− 0.091
D8S1179^a^	4.000	3.600	0.667	0.722	0.867	0.077
FGA	3.000	2.000	0.333	0.500	0.600	0.333
Penta D	2.000	1.385	0.333	0.278	0.333	− 0.200
Penta E	2.000	1.385	0.333	0.278	0.333	− 0.200
TH01^a^	4.000	3.000	1.000	0.667	0.800	− 0.500
TPOX	3.000	2.571	1.000	0.611	0.733	− 0.636
vWA^a^	4.000	3.600	0.333	0.722	0.867	0.538
Mean (± SE)	3.26 ± 0.03	2.84 ± 0.31	0.63 ± 0.07	0.57 ± 0.05	0.68 ± 0.06	− 0.13 ± 0.09

*Na* Observed number of alleles, *Ne* effective number of alleles, *Ho* observed heterozygosity, *He* expected heterozygosity, *UHe* unbiased expected heterozygosity, *F* fixation index

Loci with star (^a^) were relatively more heterozygous and were used in establishing genealogical relationship

**Table 2:** Genealogical relationship of Chimpanzees with SureID^®^ 21G and SureID^®^ 23G Human STR Identification kit (Health Gene Technologies)

SureID^®^ 21G (Health Gene Technologies) human STR Identification kit						
Chimp 1	Chimp 2	R	LnL(R)	HS	FS	PO
Chottu	Buri	U	− 62.26	7.14	15.58	9999
Mastaan	Buri	U	− 59.67	5.66	11.91	9999
Mastaan	Chottu	U	− 58.17	5.98	13.16	9999

SureID^®^ 23G Human STR Identification kit (Health Gene Technologies)						
Chimp 1	Chimp 2	R	LnL(R)	HS	FS	PO
Chottu	Buri	U	− 65.97	7.26	15.91	9999
Mastaan	Buri	U	− 71.75	7.76	16.06	9999
Mastaan	Chottu	U	− 71.46	7.98	17.10	9999

where *U* unrelated, *HS* half-siblings, *FS* full-siblings, *PO* parent-offspring

#### Applicability of SureID^®^ 23comp Human STR Identification kit

Of the 23 loci in SureID^®^ 23comp Human STR Identification kit, three loci i.e. D12S391, D8S1132, and D2S441 did not amplify in all three chimpanzee samples and other 20 loci were polymorphic. The detail allelic data can be obtained upon request. The locus AMEL further validated the known gender of chimpanzees by amplifying alleles of size 108 in female and males showing 113 and 108 bp. This validates the discrimination power of gender identification in chimpanzees using SureID^®^ 23comp Human STR Identification systems. In total, 75 alleles were found associated with 20 loci. The number of observed alleles ranged from 6 (D1S1656) to 2 (D14S1434, D19S253, D22-GATA198B05) with mean number of alleles 3.75 ± 0.27 per locus. The effective number of alleles with mean 3.31 ± 0.26 did not exceed the observed number of alleles for all loci. The mean Ho and He were 0.77 ± 0.05 and 0.66 ± 0.03 with mean UHe of 0.82 ± 0.03 (Table 3). A negative F value of − 0.17 ± 0.09 was observed that the analyzed chimpanzees plausibly belong to unrelated genetic background. Out of 20 loci, 12 loci were observed relatively more heterozygous with ≥ 4 alleles/locus indicating that the analyzed individuals were genetically unrelated. The genealogical relationship between the individuals based on four possible categories i.e. U, HS, FS and PO for 17 loci i.e. AMEL, D11S2368, D13S325, D15S659, D16S539, D18S1364, D19S253, D1S1656, D20S482, D21S2055, D3S1744, D4S2366, D5S2800, D6S474, D14S1434, D7S3048 and D9S1122 (which amplified in all three chimpanzees) suggested that analyzed individuals were genetically unrelated (LnL(R) − 71.75 to − 65.97; Table 3).

**Table 3:** Genetic diversity estimates of Chimpanzees with SureID^®^ 23comp (Health Gene Technologies) human STR Identification kit

Locus	Na	Ne	Ho	He	uHe	F
AMEL	2.000	1.800	0.667	0.444	0.533	− 0.500
D18S1364^a^	5.000	4.500	0.667	0.778	0.933	0.143
D1S1656^a^	6.000	6.000	1.000	0.833	1.000	− 0.200
D13S325^a^	4.000	3.600	0.667	0.722	0.867	0.077
D5S2800	3.000	2.571	0.667	0.611	0.733	− 0.091
D9S1122^a^	4.000	3.000	0.667	0.667	0.800	0.000
D4S2366^a^	5.000	4.500	1.000	0.778	0.933	− 0.286
D3S1744^a^	5.000	4.500	0.667	0.778	0.933	0.143
D11S2368^a^	4.000	3.600	1.000	0.722	0.867	− 0.385
D21S2055	3.000	2.571	0.667	0.611	0.733	− 0.091
D20S482^a^	4.000	3.000	1.000	0.667	0.800	− 0.500
D7S3048^a^	4.000	3.600	0.667	0.722	0.867	0.077
D19S253	2.000	1.800	0.667	0.444	0.533	− 0.500
D10S1248	3.000	2.667	1.000	0.625	0.833	− 0.600
D17S1301	3.000	2.667	1.000	0.625	0.833	− 0.600
D22-GATA198B05	2.000	2.000	1.000	0.500	1.000	− 1.000
D16S539^a^	5.000	4.500	0.667	0.778	0.933	0.143
D6S474^a^	4.000	3.000	0.667	0.667	0.800	0.000
D14S1434	2.000	1.800	0.000	0.444	0.533	1.000
D15S659^a^	5.000	4.500	1.000	0.778	0.933	− 0.286
Mean (± SE)	3.75 ± 0.27	3.31 ± 0.26	0.77 ± 0.05	0.66 ± 0.03	0.82 ± 0.03	− 0.17 ± 0.09

*Na* Observed number of alleles, *Ne* effective number of alleles, *Ho* observed heterozygosity, *He* expected heterozygosity, *UHe* unbiased expected heterozygosity, *F* fixation index

Loci with star (^a^) were relatively more heterozygous and were used in establishing genealogical relationship

### Discussion

The study showed the positive application of the *SureID*^*®*^ *21G* and *SureID*^*®*^ *23comp* STR system with Chimpanzee DNAs which can be further utilized in cases of paternity analysis, gender discrimination, population genetic studies and understanding the evolution and phylogenetic relationships among the human and non-human primates with the common marker systems. Several genetic parameters like higher observed heterozygosity than the expected heterozygosity, a negative fixation index and log-likelihood relationship index suggested that these three individuals were genetically unrelated. Time and efforts spent to explore the applicability of commercially available human STR systems *SureID*^*®*^ *21G* and *SureID*^*®*^ *23comp* STR system in Chimpanzee has been well justified and raised hope to test the applicability of these marker systems in other non-human primates like Gorilla, Orangutan, Langur and Macaques.

## Limitations

The small sample size was a limitation for this study. However present study well explains the Cross-species validation of Human specific STR systems in non human primate *(Pan Troglodytes)* and would propose further studies with large sample size.

## Data Availability

Not applicable, all relevant data is present in the manuscript.
